# A Systematic Review and Meta-Analysis of *Ginkgo biloba* in Neuropsychiatric Disorders: From Ancient Tradition to Modern-Day Medicine

**DOI:** 10.1155/2013/915691

**Published:** 2013-05-28

**Authors:** Natascia Brondino, Annalisa De Silvestri, Simona Re, Niccolò Lanati, Pia Thiemann, Anna Verna, Enzo Emanuele, Pierluigi Politi

**Affiliations:** ^1^Department of Brain and Behavioral Sciences, Section of Psychiatry, University of Pavia, Via Bassi 21, 27100 Pavia, Italy; ^2^Biometric and Statistical Unit, Fondazione IRCCS Policlinico San Matteo, Viale Golgi 2, 27100 Pavia, Italy; ^3^Department of Public Health, Neuroscience, Experimental and Forensic Medicine, Section of Psychiatry, University of Pavia, Via Bassi 21, 27100 Pavia, Italy; ^4^Psychology Institute, Ruhr-University Bochum, Universitätsstraße 150, 44801 Bochum, Germany

## Abstract

*Ginkgo biloba* (Gb) has demonstrated antioxidant and vasoactive properties as well as clinical benefits in several conditions such as ischemia, epilepsy, and peripheral nerve damage. Additionally, Gb is supposed to act as potential cognitive enhancer in dementia. So far, several trials have been conducted to investigate the potential effectiveness of Gb in neuropsychiatric conditions. However, the results of these studies remain controversial. We conducted a systematic review and a meta-analysis of three randomised controlled trials in patients with schizophrenia and eight randomised controlled trials in patients with dementia. Gb treatment reduced positive symptoms in patients with schizophrenia and improved cognitive function and activities of daily living in patients with dementia. No effect of Gb on negative symptoms in schizophrenic patients was found. The general lack of evidence prevents drawing conclusions regarding Gb effectiveness in other neuropsychiatric conditions (i.e., autism, depression, anxiety, attention-deficit hyperactivity disorder, and addiction). Our data support the use of Gb in patients with dementia and as an adjunctive therapy in schizophrenic patients.

## 1. Introduction

*Ginkgo biloba* (Gb) is one of the most ancient seed plant, often referred to as a “living fossil.” This large tree may live over 1000 years and reach 40 m of height. Originally native to China, Gb is now cultivated worldwide. Extract from Gb leaves has been used in traditional Chinese medicine for centuries to treat circulatory disorders, asthma, tinnitus, vertigo, and cognitive problems [1]. Today, Gb extracts are one of the most commonly taken phytomedicines globally [2] and are often prescribed in Europe as a nootropic agent in old age and dementia [3]. Of note, since 2000, Gb extract is included in ATC-classification as an anti-dementia drug together with cholinesterase inhibitors and memantine [4]. Gb extract contains mainly terpenoids, flavonol glycosides, and proanthocyanidins. The most prevalent of these three groups are the flavonol glycosides (quercetin, catechin). The terpenoids include ginkgolides and bilobalides, which represent unique components of Gb. Terpenoids, flavonoids and proanthocyanidins are thought to be responsible for the pharmacological properties of Gb [1]. On the basis of animal studies, several mechanisms have been proposed to explain the pharmacological properties of this plant: extract from Gb leaves inhibits platelet-activating factor [5] and enhances NO production in vessels, with subsequent effect on peripheral and cerebral blood flow [6]. Gb extract is thought to module different neurotransmitter systems: it is a strong inhibitor of monoamine oxidase A and synaptosomal uptake of DA, 5-HT, and norepinephrine [7–9]. Additionally, Gb displays a free radical scavenger activity and has neuroprotective and antiapoptotic properties, such as inhibition of amyloid-*β* neurotoxicity and protection against hypoxic challenges and increased oxidative stress [10–12]. Several previous reviews have been mainly focused on the potential efficacy of Gb in dementia. However, inconsistent and controversial results have been reported [13–16]. On the other hand, to date no systematic review has been conducted on the effect of Gb on neuropsychiatric disorders other than dementia. Therefore, we aimed to perform a systematic review on the effects of Gb in different psychiatric conditions.

## 2. Methods

In April 2012, we searched the following databases: MEDLINE, EMBASE, PsycINFO, and the Cochrane Database of Systematic Reviews. The search terms were as follows: ginkgo biloba (gingko biloba; ginkgo; ginko; gingko; bilobalid*; egb 761) and dementia (dementia OR cognitive impairment OR Alzheimer), autism (autism OR autistic spectrum disorder), schizophrenia (schizophrenia OR psychosis OR psychotic disorder OR delusion), depression (depression OR major depression OR depressive symptom), anxiety (anxiety OR generalized anxiety disorder OR anxious), attention-deficit/hyperactivity (ADHD) (attention deficit disorder OR ADHD or attention deficit OR hyperactivity), and addiction. All search terms were searched individually in each database and combined together. The search strategy had no time restriction but was limited to articles in English, Italian, French, Spanish, and German. Additionally, all recovered papers were reviewed for further relevant references. Researchers in the field were reached to obtain additional or unpublished data, if available.

We selected controlled randomized clinical trials, yielding primary results on the effects of the administration of Gb extracts in neuropsychiatric patients. Every neuropsychiatric disorder was defined according to internationally valid diagnostic criteria such as the International Classification of Diseases (ICD) or the Diagnostic and Statistical Manual of Mental Disorders (DSM). Other inclusion criteria were a minimum number of participants of ten per group, a treatment period of at least 6 weeks, and the availability of a full-text publication. Of note, all the included studies in the meta-analysis were conducted using the standardized Gb extract Egb 761, which is the most commonly used form of Gb [17].

Two researchers (NB and SR) independently reviewed all information about the articles provided by the databases. Any discrepancies were solved by consensus. We assessed the quality of the study design, duration of the study, comparability of study groups, and clinical outcomes on different widely used rating scales.

The following rating scales were accepted for clinical outcomes: (1) dementia: cognition: Syndrom-Kurz test (SKT) [18], Alzheimer's Disease Assessment Scale, cognitive subscale (ADAS-cog) [19]; activities of daily living (ADL): Alzheimer's Disease Activities-of-Daily-Living International Scale (ADL-IS) [20], Geriatric Evaluation by Relatives Rating Instrument (GERRI) [21], Gottries-Bråne-Steen-Activities of Daily Living (GBS-ADL) scale [22], Nürnberger Alters-Alltagsaktivitäten-Skala (NAA), and Nürnberger Alters-Beobachtungsskala (NAB) [23]; (2) schizophrenia: Scale for the Assessment of Positive (SAPS) [24] and Negative (SANS) Symptoms [25], Brief Psychiatric Rating Scale (BPRS) [26], (3) autism: Aberrant Behavior Checklist-Community (ABC-C) [27]; (4) Attention-Deficit/Hyperactivity Disorder (ADHD): Parent and Teacher ADHD Rating Scale-IV [28], Conners' Parent Rating Scale-Revised [29]; (5) anxiety: Hamilton Rating Scale for Anxiety (HAMA) [30], State-Trait Anxiety Inventory (STAI) [31]; and (6) tardive dyskinesia: Abnormal Involuntary Movement Scale (AIMS) [32]. 

When it was possible, data were pooled by means of meta-analysis. Effect measures on rating scales were expressed as standardized mean differences (SMDs) with the 95% CIs. A random-effects model (DerSimonian-Laird) was used to calculate a pooled effect estimate, because of heterogeneity. A *P* value <0.05 was regarded as statistically significant. Heterogeneity of effect sizes was evaluated by the *I* ^2^ statistic. An alpha error *P* < 0.05 and/or *I* ^2^ of at least 50% were taken as indicators of substantial heterogeneity of outcomes. If meta-analyses were not possible, the results of individual studies are presented. Meta-analyses were performed using Meta-Analyst and RevMan 5 for all calculations [33].

## 3. Results

**Figure 1:**
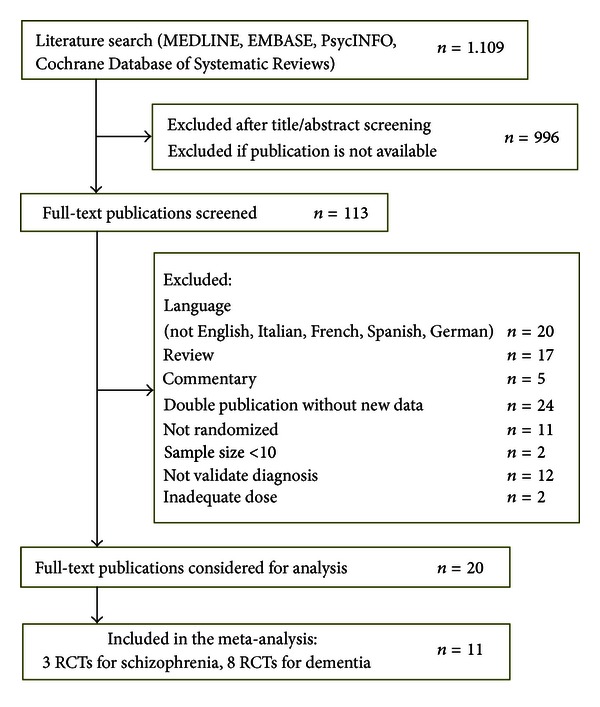
Flow chart of study selection.

**Figure 2:**
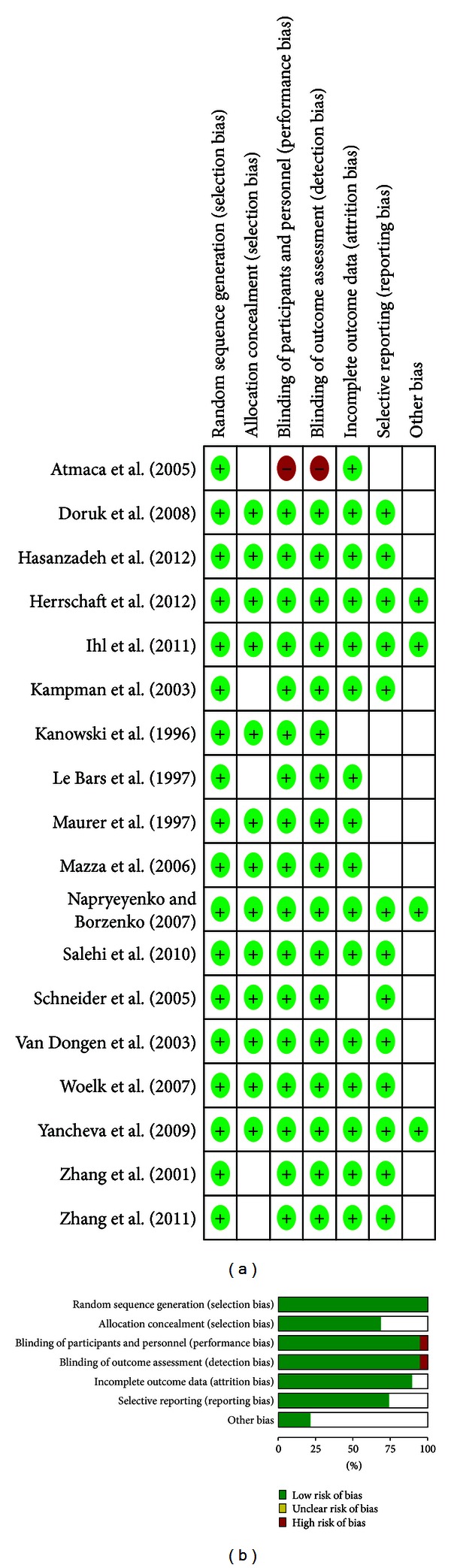
Assessment of the methodological quality of the included studies.

Our literature search identified 1109 clinical publications. After the title/abstract screening, 113 publications were obtained for detailed evaluation (Figure 1). Summary of the final articles included is shown in Table 1. Overall, the methodology of the included studies was good (Figure 2).

### 3.1. Autism

A recent study involving 47 children with a DSM-IV-TR diagnosis of autism was identified [34]. Patients were randomly assigned to receive either Gb or placebo in adjunction to risperidone. The primary outcome was the ABC-C scale. There was no statistically significant difference between the two groups according to the aforementioned subscale. Thus, Gb seemed to be not an efficacious adjunctive therapy to risperidone. However, it appeared to be safe and well tolerated even in childhood. 

### 3.2. ADHD

Salehi et al. [35] reported a double-blind trial of Gb versus methylphenidate in 50 ADHD patients. The investigators reported that Gb had no comparable efficacy in comparison with methylphenidate. Even if Gb determined significantly few side effects (especially insomnia and loss of appetite), methylphenidate determined a dramatic improvement in a range of symptoms.

### 3.3. Addiction

Only one double-blind randomized controlled study had been conducted so far involving 44 DSM-IV cocaine-dependent men and women [36]. Each participant randomly received either piracetam, Gb, or placebo. The primary outcome was the relapse from abstinence (measured as self-reported relapse, treatment dropout, or positive urine toxicology screening). At the end of the study, no significant differences were observed between the three groups.

### 3.4. Generalized Anxiety Disorder (GAD)

Only one study investigating the effects of Gb on GAD fulfilled the review criteria [37]. In 2007, 82 patients were randomly treated with Gb extract, at the dose of 480 mg/die (*n* = 27) or 240 mg/die (*n* = 25), or with placebo (*n* = 30). The primary outcome was represented by change on the HAMA scale (response defined as a reduction in HAMA total score of at least 50%). The authors reported a significant improvement in psychopathological symptoms. Response rates were 44% in the high-dose group, 31% in the low-dose group, and 22% with placebo. Additionally, the percentages of clinically significant responses were 81%, 67%, and 38% for the high-dose, the low-dose, and the placebo groups, respectively. Of note, there was a significant inverse dose-response relationship between the dose per Kg and the HAMA score. The safety of Gb extract appeared good.

### 3.5. Tardive Dyskinesia

Recently, Zhang et al. [38] evaluated Gb extract as a potential treatment for tardive dyskinesia in patients with chronic schizophrenia. One hundred and fifty-seven patients were randomized to Gb extract (*n* = 78, 240 mg/die) or placebo (*n* = 79) for 12 weeks. All participants were on antipsychotic medication (chlorpromazine equivalents were comparable between the two groups). Tardive dyskinesia severity, which represented the primary outcome of the study, was assessed by means of the Abnormal Involuntary Movement Scale (AIMS). A significant improvement was found in the Gb group in the AIMS score. It is interesting to note that, the percentage of responders (according to a decrease of at least 30% in the AIMS) was significantly higher in the treatment group (51.3% versus 5.1%). Despite the significant effect of Gb on movement symptoms, no significant effect of group was observed for psychopathological symptoms (representing a secondary outcome of the study), as both groups showed an improvement over time.

### 3.6. Schizophrenia

**Figure 3:**
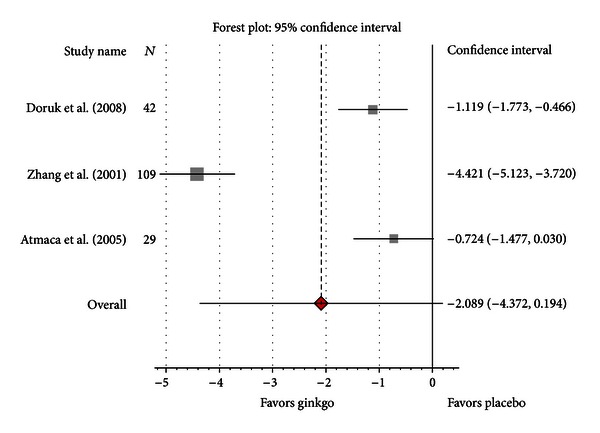
Pooled standardized mean difference compared with placebo for negative symptoms score (SANS).

Three randomized clinical trials evaluating Gb extract in patients with schizophrenia were included in the analysis [39–41]. Two studies were double-blind and placebo controlled. Randomization procedure and methodology were considered adequate in all cases. Gb was used as an adjunctive therapy to different antipsychotics: clozapine (Doruk et al.) [39], haloperidol (Zhang et al.) [40], and olanzapine (Atmaca et al.) [41]. Mean chlorpromazine equivalent doses were comparable in the first two studies (8.3 and 8.4, resp.), while the third one used lower chlorpromazine equivalent doses (3.3). All studies included only patients with chronic schizophrenia. All three trials used SANS and SAPS (ratings for this scale were however available only in two studies) as outcome measures for clinical improvement. Change scores for SANS ranged from −7.9 to −3.5 in the Gb groups and from −2.7 to 5.3 in the placebo groups, whereas change scores for SAPS ranged from −9.4 to −4.3 in the Gb groups and from −3.8 to −0.7 in the placebo groups. Standardized mean differences for SANS score were greater for Gb than for placebo, with SMD = −2.09 (95% CI −4.34; 0.148, *H* = 5.52) (Figure 3) but not significant. Heterogeneity was substantial (*I* ^2^ = 97%). To perform sensitivity analysis, we decided to remove the study from Atmaca et al. which used lower chlorpromazine equivalent, in order to determine the impact of this trial on the results. Removing this trial did not significantly change our findings. After excluding this study, the SMD for negative symptomatology was −2.74 (95% CI −5.97; 0.48, *P* = 0.10). Heterogeneity remained substantial (*I* ^2^ = 98%).

**Figure 4:**
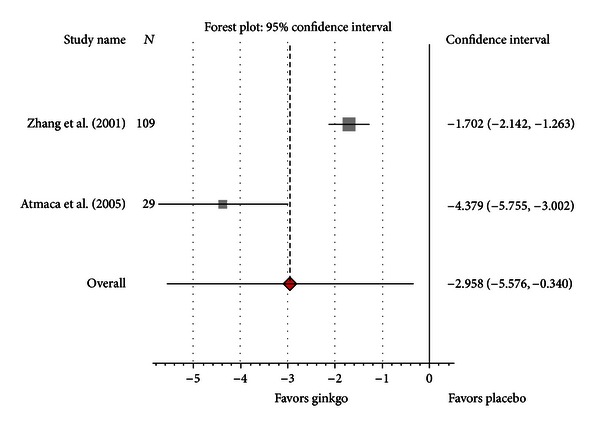
Pooled standardized mean difference compared with placebo for positive symptoms score (SAPS).

For SAPS, standardized change scores were significantly greater for Gb than for placebo, with SMD = −2.89 (95% CI −5.39; −0.38, *H* = 3.46, *P* = 0.001) (Figure 4). Heterogeneity was substantial (*I* ^2^ = 92%).

### 3.7. Dementia

**Figure 5:**
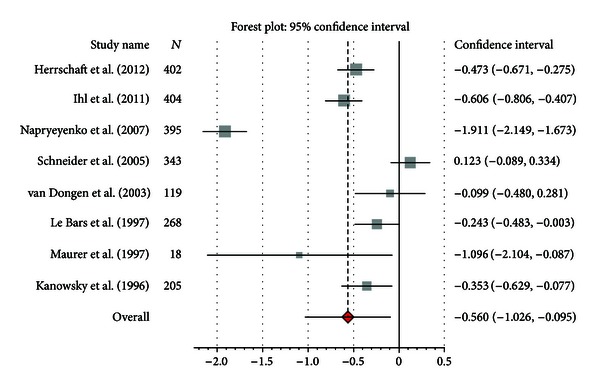
Pooled standardized mean difference compared with placebo for cognitive outcomes (ADAS-cog, SKT).

Ten studies fulfilled the inclusion criteria: meta-analysis was performed only on eight studies [42–49] which were comparable for clinical purposes. Eight studies were placebo controlled, while two studies were a head-to-head trial with donepezil as comparison group [50] or a triple-blind study with Gb, donepezil, and placebo [51]. The very different dosages of Gb and donepezil rendered meta-analytical examination unfeasible in the latter studies. All studies were randomized, double-blind trials. Overall, the methodological quality of the included studies was judged as adequate, with most studies using an intent-to-treat analysis. All studies considered the administration of a standardized extract (EGb761) in patients with Alzheimer's disease, but some sample groups also included patients with vascular dementia. In all included trials a standardized extract (EGb761) was used. For meta-analysis, we focused on the effect of Gb on cognition and ADL. Cognition was measured in two studies with the ADAS-cog [44, 47], whereas in the remaining six studies the SKT was applied. Mean differences for ADAS-cog varied between −0.3 and 1.3 in the Gb groups and from 0.9 to 1.0 in the placebo groups. Change scores in SKT ranged from −3.2 to −0.8 in Gb treated patients and from −1.2 to 1.3 in the placebo groups. Standardized mean differences were higher for Gb than for placebo, with SMD = −0.56 (95% CI −1.026; −0.095, *P* = 0.001) (Figure 5). Of note, heterogeneity was substantial (*I* ^2^ = 96,1%). If only studies using SKT were considered, we still observed an advantage for Gb compared to placebo, with SMD = −0.72 (95% CI −1.28; −0.017, *P* = 0.001): heterogeneity remained substantial (*I* ^2^ = 96%). If we considered studies using ADAS-cog, Gb was not different from placebo, with SMD = −0.05 (95% CI −0.41; 0.30, *P* = ns). Heterogeneity remained substantial (*I* ^2^ = 81%). To perform sensitivity analysis, we tried to remove the older trials in which the quality of methodological design was not as high as in most recent studies. After excluding these trials [47–49], our results did not significantly change (SMD = −0.49 (95% CI −0.59; −0.40), *P* = 0.001); of note, heterogeneity became higher (*I* ^2^ = 98%). 

**Figure 6:**
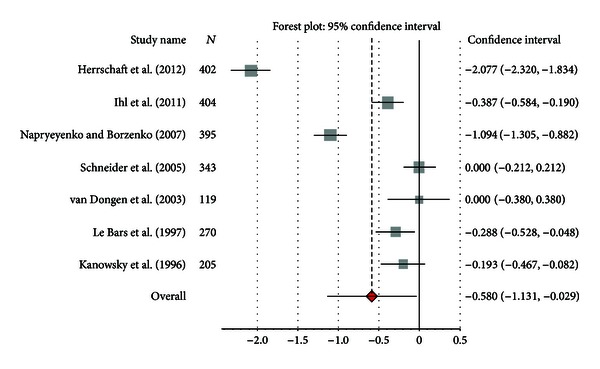
Pooled standardized mean difference compared with placebo for activities of daily living outcomes (ADL-IS, GERRI, GBS-ADL, NAA, and NAB).

ADLs were measured with different scales. Two studies used the ADL-IS [42, 43], two studies used the GERRI [47, 49], one study used the GBS-ADL subscale [44], one study used the Nürnberger Alters-Alltagsaktivitäten-skala (NAA, self-assessed) [46], and one trial used the Nürnberger Alters-Beobachtungsskala (NAB, caregiver rated) [49]. Mean differences varied in the Gb and the placebo groups between −1.9 and −0.05 and between −0.4 and 0.9, respectively. There was a significant difference in ADL standardized change scores between Gb and placebo, with SMD = −0.598 (95% CI −0.954; −0.251, *P* = 0.001) (Figure 6). Of note, we found substantial heterogeneity (*I* ^2^ = 98%). If only studies using the same scale were pooled together, we still observed a difference between Gb and placebo, favouring Gb, for the ADL-IS (SMD = −1.06 (95% CI −1.21; −0.90), *P* = 0.001) (*I* ^2^ = 99%). No difference between the two groups was observed for the GERRI (SMD = −0.04 (95% CI −0.10; 0.02), *P* = 0.15) (*I* ^2^ = 72%). The two trials performing a comparison between Gb and donepezil reported no statistically significant differences between the cholinesterase inhibitor and Gb in treating mild to moderate dementia. Both studies showed comparable treatment time, but the study of Ihl et al. [52] used significantly lower dose of both donepezil (5 mg instead of 10 mg/die) and Gb (160 mg versus 240 mg/die).

## 4. Discussion

The effect of *Ginkgo biloba* has been studied in a variety of neuropsychiatric conditions. However, the general lack of evidence prevents drawing conclusions regarding Gb effectiveness in many neuropsychiatric conditions, such as autism, ADHD, addiction, GAD, and tardive dyskinesia. Of all the psychiatric disorders reviewed, dementia has been the most extensively studied. Our meta-analysis of eight studies in dementia showed that Gb differed significantly from placebo, providing beneficial effects both in cognition and activities of daily living. Our results are consistent with a recent meta-analysis [13] on the effect of Gb on cognition. On the other hand, we found a significant difference between Gb and placebo for activities of daily living in patients with dementia which were not significant in the aforementioned report [13]. This difference may be at least in part due to the inclusion of a very recent study, yielding significant positive results in this area of functioning. We decided to pool together studies using different scales evaluating the same domain (i.e., SKT and ADAS-cog for cognition). Considering cognition, it has been reported that both ADAS-cog and SKT could be statistically compared [52]. Additionally, even if we separated the two scales, the beneficial effect of Gb remained evident at least for the SKT. Of note, we did not observe a significant improvement in heterogeneity. Considering the activities of daily living domain, there is a lack of studies using the same outcome scale; thus, we pooled together different questionnaires (measuring the same area) in order to improve power. However, if we considered only trials using the same outcome scale, we still observed a beneficial effect of Gb in the ADL-IS. Although there is clear heterogeneity, we were unable to explain it. Sensitivity analysis excluding trial with poorer methodological quality did not explain the heterogeneity. Under these circumstances, we dealt with the existence of heterogeneity using a random-effect model. 

Notwithstanding the shortage of specific studies, available evidence also supports the use of Gb in chronic schizophrenia. In particular, Gb seems to exert a beneficial effect on positive psychotic symptoms. No significant effect on negative symptoms has been observed. Even if the three included studies were similar in design (inclusion/exclusion criteria, time, and Gb dosage), all patients were on antipsychotic medication. In particular, we performed sensitivity analysis excluding one study with different chlorpromazine equivalents. In fact, the study from Atmaca et al. used a lower dosage of chlorpromazine equivalent, even if the mean dose (16.8 mg/day) of the administered drug (olanzapine) was clinically appropriate. However, heterogeneity was not modified. 

The beneficial effect of Gb in both dementia and chronic schizophrenia is however modest. Particularly, the mean effect observed in cognition is sometimes lower than what is considered clinically meaningful [52]. However, Gb was equal to donepezil in two recent clinical trials, thus potentially providing an evidence for its use in dementia, which to date could be treated with few pharmacological agents. Of note, Gb is generally used as an adjunctive therapy in schizophrenia, not as a first-line intervention, and, thus, even a small additional improvement could be valuable. Notably, all trials demonstrated an excellent safety profile for Gb.

Limitations should caution against overinterpretation of the findings. The included studies showed high heterogeneity, which could possibly have biased our results. Additionally, whether longer trials would yield more significant results in dementia and schizophrenia remains to be seen. Another potential limitation is that even though our search was systematic and rigorous, we could have missed eligible studies inadvertently.

## 5. Conclusion

Despite the heterogeneity of the clinical trials, available evidence is sufficient to support the use of Gb in patients with dementia and as an adjunctive therapy in schizophrenic patients. Despite the promising results, broad recommendations for the use of Gb in other neuropsychiatric conditions, such as ADHD, autism, and AD are still premature. A better understanding of the mechanisms of Gb effect in these conditions may be useful as well as linking Gb beneficial effects with other types of data such as fMRI or SPECT imaging. It should be considered to run major multicenter studies in order to shed more light on the effectiveness of Gb in dementia subgroups and schizophrenia. Hopefully, the design of the study should use currently available level of treatment and care, in order to provide a broader generalizability of the results.

## Figures and Tables

**Table 1:** General characteristics of the included studies.

Authors	Year	Gb dose	Type of study	Comparator	Concomitant medications	Outcome measure	Findings
Attention-deficit and hyperactivity disorder (ADHD)							

Salehi et al. [35]	2010	80 mg/day if weight <30 kg; otherwise 120 mg/day	Randomized, 6 week	Methylphenidate 20 mg/day if weight <30 kg; otherwise 30 mg/day	None	Parent and Teacher ADHD Rating Scale-IV	Significant improvement with methylphenidate

Autism							

Hasanzadeh et al. [34]	2012	80 mg/day if weight <30 kg; otherwise 120 mg/day	Randomized placebo controlled, 10 weeks	Placebo	Risperidone 2-3 mg/day according to weight	ABC-C	No difference

Cocaine addiction							

Kampman et al. [36]	2003	240 mg/day	Randomized placebo controlled, 10 weeks	Piracetam 4.8 g/day or placebo	None	Urine toxicology screen or self-report relapse	No significant difference of both piracetam or Gb to placebo

Dementia							

Herrschaft et al. [42]	2012	240 mg/day	Randomized placebo controlled, 24 weeks	Placebo	Antihypertensive, antithrombotic drug	SKT, NPI, AD CGI, ADL, QOL	Significant improvement with active treatment
Ihl et al. [43]	2011	240 mg/day	Randomized placebo controlled, 24 weeks	Placebo	Antihypertensive, antithrombotic drug	SKT, NPI, AD CGI, ADL, QOL	Significant improvement with active treatment
Napryeyenko and Borzenko [44]	2007	240 mg/day	Randomized placebo controlled, 22 weeks	Placebo	Antihypertensive, antithrombotic drug	SKT, NPI, ADL	Significant improvement
Schneider et al. [45]	2005	120 or 240 mg/day	Randomized placebo controlled, 26 weeks	Placebo		ADAS-cog	Improvement
van Dongen et al. [46]	2003	160 or 240 mg/day	Randomized placebo controlled, 24 weeks	Placebo		SKT, CGI, NAI-NAA	No differences between Gb and placebo
Le Bars et al. [47]	1997	120 mg/day	Randomized placebo controlled, 52 weeks	Placebo		ADAS-Cog, GERRI, CGIC	Significant improvement in ADAS-cog and GERRI
Maurer et al. [48]	1997	240 mg/day	Randomized placebo controlled, 12 weeks	Placebo		SKT, ADAS-cog, CGI	Significant improvement in SKT
Kanowski et al. [49]	1996	240 mg/day	Randomized placebo controlled, 24 weeks	Placebo		SKT, CGI, NBA	Significant improvement
Yancheva et al. [50]	2009	240 mg/day	Randomized versus donepezil or Gb and donepezil, 22 weeks	Donepezil 10 mg/day	Antihypertensive, antithrombotic drug	SKT, NPI, ADL	No significant differences between treatments
Mazza et al. [51]	2006	160 mg/day	Randomized placebo controlled, double blind, 24 weeks	Donepezil 10 mg/day	Benzodiazepines or antipsychotics at low dosage	MMSE, SKT, CGI	Significant improvement compared to placebo, no differences with donepezil

Generalized anxiety disorder (GAD)							

Woelk et al. [37]	2007	Two groups: low dose 240 mg/day; high dose 480 mg/day	Randomized placebo controlled, 4 weeks	Placebo	None	HAMA scale	Significant improvement compared to placebo, dose-response relationship

Schizophrenia							

Doruk et al. [39]	2008	120 mg/day	Randomized placebo controlled, 12 weeks	Placebo	Clozapine 350–500 mg/day	SANS, SAPS, BPRS	Significant improvement in negative symptoms with Gb
Zhang et al. [40]	2001	360 mg/day	Randomized placebo controlled, 12 weeks	Placebo	Haloperidol 0.25 mg/kg/day	SANS, SAPS, BPRS	Significant improvement in positive symptoms and negative symptoms with Gb
Atmaca et al. [41]	2005	300 mg/day	Randomized olanzapine and EGb versus olanzapine alone, 8 weeks	Placebo	Olanzapine 5–20 mg/day	SANS, SAPS	Significant improvement in positive symptoms with Gb

Tardive dyskinesia							

Zhang et al. [38]	2011	240 mg/day	Randomized placebo controlled, 12 weeks	Placebo	Antipsychotic or cholinergic agents	AIMS and SANS and SAPS	Significant change in AIMS score. No effect of Gb on psychopathological symptoms

ADHD: attention-deficit hyperactivity disorder; GAD: generalized anxiety disorder; ABC-C: Aberrant Behavior Checklist-Community; HAMA scale: Hamilton Rating Scale for Anxiety; AIMS: Abnormal Involuntary Movement Scale; SANS: Scale for the Assessment of Negative Symptoms; SAPS: Scale for the Assessment of Positive Symptoms; BPRS: Brief Psychiatric Rating Scale; SKT: Syndrom-Kurz test; NPI: Neuropsychiatric Inventory; AD CGI: Clinical Global Impressions Severity of AD; ADL: activities of daily living; QOL: quality of life; ADAS-cog: Alzheimer's Disease Assessment Scale-cognitive subscale; NAI-NAA: Nürnberger Alters Inventar-Nürnberger Alters-Alltagsaktivitäten-Skala; NAB: Nürnberger Alters-Beobachtungsskala; GERRI: Geriatric Evaluation by Relatives Rating Instrument; MMSE: Mini-Mental State Examination.

## References

[1] Kleijnen J, Knipschild P (1992). Ginkgo biloba. *The Lancet*.

[2] Ernst E (2002). The risk-benefit profile of commonly used herbal therapies: Ginkgo, St. John’s wort, ginseng, echinacea, saw palmetto, and kava. *Annals of Internal Medicine*.

[3] Kennedy DO, Wightman EL (2011). Herbal extracts and phytochemicals: plant secondary metabolites and the enhancement of human brain function. *Advances in Nutrition*.

[4] (2013). *ATC/DDD Index 2013*.

[5] Koch E (2005). Inhibition of platelet activating factor (PAF)-induced aggregation of human thrombocytes by ginkgolides: considerations on possible bleeding complications after oral intake of Ginkgo biloba extracts. *Phytomedicine*.

[6] Koltermann A, Hartkorn A, Koch E, Fürst R, Vollmar AM, Zahler S (2007). Ginkgo biloba extract EGb 761 increases endothelial nitric oxide production in vitro and in vivo. *Cellular and Molecular Life Sciences*.

[7] Ramassamy C, Christen Y, Clostre F, Costentin J (1992). The Ginkgo biloba extract, EGb761, increases synaptosomal uptake of 5-hydroxytryptamine: In-vitro and ex-vivo studies. *Journal of Pharmacy and Pharmacology*.

[8] Yoshitake T, Yoshitake S, Kehr J (2010). The Ginkgo biloba extract EGb 761 and its main constituent flavonoids and ginkgolides increase extracellular dopamine levels in the rat prefrontal cortex: RESEARCH PAPER. *British Journal of Pharmacology*.

[9] Fehske CJ, Leuner K, Müller WE (2009). Ginkgo biloba extract (EGb761) influences monoaminergic neurotransmission via inhibition of NE uptake, but not MAO activity after chronic treatment. *Pharmacological Research*.

[10] Wu Y, Wu Z, Butko P (2006). Amyloid-*β*-induced pathological behaviors are suppressed by Ginkgo biloba extract EGB 761 and ginkgolides in transgenic Caenorhabditis elegans. *Journal of Neuroscience*.

[11] Bastianetto S, Ramassamy C, Doré S, Christen Y, Poirier J, Quirion R (2000). The ginkgo biloba extract (EGb 761) protects hippocampal neurons against cell death induced by *β*-amyloid. *European Journal of Neuroscience*.

[12] Kampkötter A, Pielarski T, Rohrig R (2007). The Ginkgo biloba extract EGb761 reduces stress sensitivity, ROS accumulation and expression of catalase and glutathione S-transferase 4 in Caenorhabditis elegans. *Pharmacological Research*.

[13] Weinmann S, Roll S, Schwarzbach C, Vauth C, Willich SN (2010). Effects of Ginkgo biloba in dementia: systematic review and meta-analysis. *BMC Geriatrics*.

[14] Birks J, Evans JG (2009). Ginkgo biloba for cognitive impairment and dementia. *Cochrane Database of Systematic Reviews*.

[15] Kasper S, Schubert H (2009). Ginkgo-spezialextrakt EGb 761 in der behandlung der demenz: evidenz für wirksamkeit und verträglichkeit. *Fortschr Neurol Psychiatr*.

[16] Hoerr R (2003). Behavioural and psychological symptoms of dementia (BPSD): effects of EGb 761. *Pharmacopsychiatry*.

[17] Birks J, Grimley EV, Van Dongen M (2002). Ginkgo biloba for cognitive impairment and dementia. *Cochrane Database of Systematic Reviews*.

[18] Kim YS, Nibbelink DW, Overall JE (1993). Factor structure and scoring of the SKT test battery. *Journal of Clinical Psychology*.

[19] Rosen WG, Mohs RC, Davis KL (1984). A new rating scale for Alzheimer’s disease. *American Journal of Psychiatry*.

[20] Reisberg B, Finkel S, Overall J (2001). The Alzheimer’s disease activities of daily living international scale (ADL-IS). *International Psychogeriatrics*.

[21] Schwartz GE (1983). Development and validation of the geriatric evaluation by relative’s rating instrument (GERRI). *Psychological Reports*.

[22] Bråne G, Gottfries CG, Winblad B (2001). The Gottfries-Bråne-Steen scale: validity, reliability and application in anti-dementia drug trials. *Dementia and Geriatric Cognitive Disorders*.

[23] Oswald WD, Fleischmann UM (1995). *Nürnberger-Alters-Inventar (NAI)-Testmanual Und Textband Göttingen*.

[24] Andreasen NC (1984). *The Scale For the Assessment of Positive Symptoms (SAPS)*.

[25] Andreasen NC (1989). The scale for the assessment of negative symptoms (SANS): conceptual and theoretical foundations. *The British Journal of Psychiatry*.

[26] Overall JE, Gorham DR (1962). The brief psychiatric rating scale. *Psychological Reports*.

[27] Aman MG, Singh NN, Stewart AW, Field CJ (1985). The aberrant behavior checklist: a behavior rating scale for the assessment of treatment effects. *American Journal of Mental Deficiency*.

[28] DuPaul G, Power T, Anastopoulos A, Reid R (1998). *ADHD Rating Scale-IV*.

[29] Conners CK (1997). *Conners' Rating Scales—Revised*.

[30] Hamilton M (1959). The assessment of anxiety states by rating. *British Journal of Medical Psychology*.

[31] Spielberger CD, Gorssuch RL, Lushene PR, Vagg PR, Jacobs GA (1970). *Manual for the State-Trait Anxiety Inventory*.

[32] Schooler NR, Kane JM (1982). Research diagnoses for tardive dyskinesia. *Archives of General Psychiatry*.

[33] Wallace BC, Schmid CH, Lau J, Trikalinos TA (2009). Meta-analyst: software for meta-analysis of binary, continuous and diagnostic data. *BMC Medical Research Methodology*.

[34] Hasanzadeh E, Mohammadi MR, Ghanizadeh A (2012). A double-blind placebo controlled trial of Ginkgo biloba added to risperidone in patients with autistic disorders. *Child Psychiatry & Human Development*.

[35] Salehi B, Imani R, Mohammadi MR (2010). Ginkgo biloba for attention-deficit/hyperactivity disorder in children and adolescents: a double blind, randomized controlled trial. *Progress in Neuro-Psychopharmacology and Biological Psychiatry*.

[36] Kampman K, Majewska MD, Tourian K (2003). A pilot trial of piracetam and ginkgo biloba for the treatment of cocaine dependence. *Addictive Behaviors*.

[37] Woelk H, Arnoldt KH, Kieser M, Hoerr R (2007). Ginkgo biloba special extract EGb 761 in generalized anxiety disorder and adjustment disorder with anxious mood: a randomized, double-blind, placebo-controlled trial. *Journal of Psychiatric Research*.

[38] Zhang WF, Tan YL, Zhang XY, Chan RCK, Wu HR, Zhou DF (2011). Extract of Ginkgo biloba treatment for tardive dyskinesia in schizophrenia: a randomized, double-blind, placebo-controlled trial. *Journal of Clinical Psychiatry*.

[39] Doruk A, Uzun O, Özşahin A (2008). A placebo-controlled study of extract of ginkgo biloba added to clozapine in patients with treatment-resistant schizophrenia. *International Clinical Psychopharmacology*.

[40] Zhang XY, Zhou DF, Zhang PY, Wu GY, Su JM, Cao LY (2001). A double-blind, placebo-controlled trial of extract of Ginkgo biloba added to haloperidol in treatment-resistant patients with Schizophrenia. *Journal of Clinical Psychiatry*.

[41] Atmaca M, Tezcan E, Kuloglu M, Ustundag B, Kirtas O (2005). The effect of extract of ginkgo biloba addition to olanzapine on therapeutic effect and antioxidant enzyme levels in patients with schizophrenia. *Psychiatry and Clinical Neurosciences*.

[42] Herrschaft H, Nacu A, Likhachev S, Sholomov I, Hoerr R, Schlaefke S (2012). Ginkgo biloba extract EGb 761 in dementia with neuropsychiatric features: a randomised, placebo-controlled trial to confirm the efficacy and safety of a daily dose of 240 mg. *Journal of Psychiatric Research*.

[43] Ihl R, Bachinskaya N, Korczyn AD (2011). Efficacy and safety of a once-daily formulation of Ginkgo biloba extract EGb 761 in dementia with neuropsychiatric features: a randomized controlled trial. *International Journal of Geriatric Psychiatry*.

[44] Napryeyenko O, Borzenko I, GINDEM-NP Study Group (2007). Ginkgo biloba special extract in dementia with neuropsychiatric features: a randomised, placebo-controlled, double-blind clinical trial. *Arzneimittel-Forschung/Drug Research*.

[45] Schneider LS, DeKosky ST, Farlow MR, Tariot PN, Hoerr R, Kieser M (2005). A randomized, double-blind, placebo-controlled trial of two doses of Ginkgo biloba extract in dementia of the Alzheimer’s type. *Current Alzheimer Research*.

[46] van Dongen M, van Rossum E, Kessels A, Sielhorst H, Knipschild P (2003). Ginkgo for elderly people with dementia and age-associated memory impairment: a randomized clinical trial. *Journal of Clinical Epidemiology*.

[47] Le Bars PL, Katz MM, Berman N, Itil TM, Freedman AM, Schatzberg AF (1997). A placebo-controlled, double-blind, randomized trial of an extract of Ginkgo biloba for dementia. *Journal of the American Medical Association*.

[48] Maurer K, Ihl R, Dierks T, Frölich L (1997). Clinical efficacy of Ginkgo biloba special extract EGb 761 in dementia of the Alzheimer type. *Journal of Psychiatric Research*.

[49] Kanowski S, Herrmann WM, Stephan K, Wierich W, Hörr R (1996). Proof of efficacy of the ginkgo biloba special extract EGb 761 in outpatients suffering from mild to moderate primary degenerative dementia of the Alzheimer type or multi-infarct dementia. *Pharmacopsychiatry*.

[50] Yancheva S, Ihl R, Nikolova G, Panayotov P, Schlaefke S, Hoerr R (2009). Ginkgo biloba extract EGb 761, donepezil or both combined in the treatment of Alzheimer’s disease with neuropsychiatric features: a randomised, double-blind, exploratory trial. *Aging and Mental Health*.

[51] Mazza M, Capuano A, Bria P, Mazza S (2006). Ginkgo biloba and donepezil: a comparison in the treatment of Alzheimer’s dementia in a randomized placebo-controlled double-blind study. *European Journal of Neurology*.

[52] Ihl R, Grass-Kapanke B, Jänner M, Weyer G (1999). Neuropsychometric tests in cross sectional and longitudinal studies—a regression analysis of ADAS-cog, SKT and MMSE. *Pharmacopsychiatry*.

